# A Membrane‐Centric Plasma Lipidomic Signature of Response to Long‐Acting Naltrexone in Alcohol Use Disorder

**DOI:** 10.1111/adb.70165

**Published:** 2026-05-12

**Authors:** Liyao Huang, Bojie Zhou, Qinling Ou, Jubo Yin, Xiwen Tian, Xuhui Zhou

**Affiliations:** ^1^ The School of Clinical Medicine Hunan University of Chinese Medicine Changsha Hunan China; ^2^ Department of Addiction Medicine Hunan Institute of Mental Health, The Second People's Hospital of Hunan Province (Brain Hospital of Hunan Province) Changsha Hunan China; ^3^ Fuzhou Neuropsychiatric Hospital Affiliated to Fujian Medical University Fuzhou Fujian China; ^4^ The Second General Hospital of Fuzhou Neuropsychiatric Prevention and Treatment Hospital Fuzhou Fujian China; ^5^ College of Integrated Chinese and Western Medicine Hunan University of Chinese Medicine Changsha Hunan China

**Keywords:** alcohol use disorder, lipidomics, naltrexone implant, PC/PE ratio, phospholipid remodelling

## Abstract

Long‐acting naltrexone is a first‐line pharmacotherapy for alcohol use disorder (AUD), but clinical response is heterogeneous and the underlying biology remains incompletely understood. In a randomised, double‐blind, placebo‐controlled trial of naltrexone implants (*n* = 70), the prespecified primary endpoint—percentage of heavy‐drinking days (PHDD) over 24 weeks—favoured naltrexone (median 1.49% vs. 13.39%; *p* = 0.042). In a prespecified Week‐12 mechanistic lipidomics substudy, untargeted liquid chromatography–mass spectrometry (LC–MS) profiling was performed in a responder‐enriched subset comprising naltrexone responders (RN, *n* = 18), placebo‐treated participants (PL, *n* = 10) and age‐ and BMI‐matched healthy male controls (HC, *n* = 10). We derived membrane‐related indices reflecting two prespecified axes of phospholipid remodelling: headgroup balance (PC/PE) and acyl‐chain composition (arachidonic acid [AA] and n‐3 polyunsaturated fatty acids within phospholipid pools). RN showed higher PC/PE and coordinated acyl‐chain shifts versus PL, with species‐level changes preferentially moving toward the healthy‐control direction. Exploratory analyses in naltrexone nonresponders (NR, *n* = 10) revealed partial Lands‐cycle‐related shifts but lacked the broader dual‐axis configuration observed in RN. In RN + PL (*n* = 28), higher n‐3 in PE and lower AA in PE at Week 12 were prospectively associated with greater subsequent heavy‐drinking burden. These findings support a dual‐axis membrane remodelling phenotype associated with naltrexone response and prospectively linked to heavy‐drinking burden in AUD, providing a biologically grounded framework for future mechanistic and biomarker studies.

## Graphical abstract

In this responder‐enriched lipidomic substudy, response to long‐acting naltrexone in alcohol use disorder was associated with coordinated remodelling of plasma membrane phospholipids. The signature involved PEMT‐related PC/PE shifts, Lands‐cycle acyl chain editing, and cross‐headgroup redistribution of n‐3 PUFAs and arachidonic acid, defining a membrane‐centric phenotype linked to reduced heavy drinking burden.

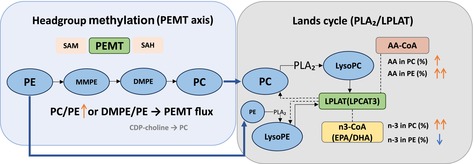

## 1 Introduction

Alcohol use disorder (AUD) is a chronic, relapsing brain condition marked by compulsive alcohol seeking, impaired control over use and withdrawal on cessation. Pathophysiologically, dysregulation of neural circuits governing reward, motivation and memory drives continued alcohol use despite adverse consequences. Globally, AUD constitutes a substantial public health burden, contributing significantly to morbidity and mortality. While prevalence rates have historically varied by region, the clinical imperative remains constant: the need for effective therapeutic strategies to sustain abstinence and prevent the cycle of relapse [1, 2, 3].

Relapse remains a principal barrier to sustained recovery. After inpatient or medically supervised detoxification, approximately half to two‐thirds of patients relapse within the first 6 months [4, 5, 6]. Repeated relapse–abstinence cycles are associated with delayed cognitive recovery and greater impulsivity, which undermine adherence and increase morbidity and mortality [7, 8, 9]. Standard care combines pharmacotherapy with psychosocial interventions [10]. For relapse prevention, widely used medications include disulfiram, acamprosate and naltrexone. Naltrexone—a μ‐opioid receptor antagonist with weaker κ and δ antagonism—blunts alcohol‐related reward signalling and is recommended as a first‐line option for moderate‐to‐severe AUD [10, 11]. Long‐acting formulations, including implants and extended‐release injections, have been developed to improve adherence and stabilise therapeutic plasma levels [12, 13, 14] . Nonetheless, meta‐analyses show small‐to‐moderate average effects with substantial heterogeneity, consistent with multi‐system mechanisms [11, 15, 16, 17] . This variability suggests that the mechanisms underlying successful treatment response may involve physiological processes that extend beyond simple receptor occupancy, potentially engaging broader metabolic and homeostatic systems.

Identifying molecular correlates of treatment response is critical for advancing precision medicine in AUD. While neuroimaging studies have documented widespread white matter microstructural abnormalities in AUD—suggestive of compromised membrane integrity—systemic metabolic markers reflecting these membrane alterations remain underutilised [18, 19]. In line with this, we previously observed mTORC1‐linked pathway abnormalities in an integrated metabolomic–lipidomic study of AUD with cognitive impairment [20]. Together, these observations converge on lipid dysregulation as a disease‐relevant feature. Alcohol exerts profound metabolic stress on lipid homeostasis, particularly affecting biological membranes. Two fundamental axes of membrane remodelling are particularly relevant: (i) headgroup methylation via phosphatidylethanolamine *N*‐methyltransferase (PEMT), a pathway known to be impaired by alcohol‐induced metabolic stress [21], and (ii) acyl‐chain editing through the Lands cycle, mediated by phospholipase A_2_ (PLA_2_) and lysophosphatidylcholine acyltransferase (LPCAT3) [22, 23].

These two axes are functionally relevant because they shape complementary aspects of membrane state. Changes in headgroup balance, reflected by phosphatidylcholine‐to‐phosphatidylethanolamine (PC/PE), influence bilayer packing, curvature stress, membrane integrity and organelle homeostasis [24]. In parallel, the distribution of AA and n‐3 PUFAs across phospholipid pools reflects acyl‐chain remodelling, with potential consequences for membrane fluidity, signalling precursor availability [25] and membrane‐associated signalling. Accordingly, these indices are not merely compositional readouts, but biologically meaningful markers of membrane state that may be relevant to reward processing and relapse vulnerability in AUD [26]. Yet, lipid change during recovery from AUD should not be assumed to represent uniform normalisation. Available evidence suggests that abstinence‐related lipid shifts are often selective and incomplete rather than global [27, 28]. It therefore remains unclear whether pharmacological treatment with naltrexone is associated with a membrane‐remodelling pattern that is distinct from, and potentially more organised than, lipid changes related to abstinence alone. Disentangling treatment‐associated lipidomic signatures from nonspecific abstinence‐related change thus requires a placebo (PL)‐controlled design, which the present study was designed to provide.

In the present study, we conducted a randomised, double‐blind, PL‐controlled clinical trial of a long‐acting naltrexone implant. We employed untargeted plasma lipidomics at Week 12 to characterise the phospholipid profiles of naltrexone responders (RN) compared with PL‐treated participants (PL) and healthy controls (HC). We utilised a responder‐enrichment analysis strategy to maximise the detection of treatment‐associated biological signals. Our analysis focused on prespecified systems‐level indices spanning the PEMT and Lands‐cycle axes, specifically the PC/PE ratio and the distribution of arachidonic acid (AA) and n‐3 polyunsaturated fatty acids. We hypothesised that naltrexone response would be characterised by a coherent dual‐axis membrane remodelling signature—spanning headgroup balance (PC/PE) and Lands‐cycle‐linked PUFA partitioning—that is distinct from lipid changes attributable to abstinence alone. Furthermore, we examined the prospective association between these Week 12 membrane indices and subsequent drinking outcomes to assess their potential prognostic value.

## 2 Methods

### 2.1 Trial Design and Endpoints

A randomised, double‐blind, PL‐controlled, single‐centre trial was conducted at the Addiction Medicine Center of Hunan Second People's Hospital (April 2023 to October 2024). The trial was registered in the Chinese Clinical Trial Registry (CTR20222972). Participants were recruited from both outpatient and inpatient settings. Before enrolment, all participants had completed detoxification treatment and were clinically stable prior to randomisation and implant administration.

Eligible participants were adults aged 18 years or older who met DSM‐5 criteria for moderate‐to‐severe AUD, had completed detoxification, had remained clinically stable without significant withdrawal symptoms before randomisation, were able to provide recent Timeline Follow‐Back (TLFB) drinking data and had a recent history of repeated heavy drinking. Key exclusion criteria included pregnancy or lactation, clinically significant hepatic dysfunction, uncontrolled active infection, severe uncontrolled systemic or psychiatric illness, recent anti‐relapse treatment, opioid use or anticipated opioid requirement during the study and other conditions compromising safety or trial participation. Detailed eligibility criteria are provided in the Supplementary Methods.

Participants were randomised via an Interactive Web Response System (IWRS) to receive a subcutaneous naltrexone implant (0.9 or 1.5 g) or a matched PL implant. For the present analyses, the two active‐dose groups were pooled and analysed together as the naltrexone‐implant (NI) group (*n* = 47), while 23 participants received placebo (PL). Randomisation was not stratified by baseline variables. Participants, investigators and outcome assessors remained blinded for 24 weeks.

Daily drinking data were collected using the TLFB method, based on daily drinking record forms completed by participants and their family members. The prespecified primary endpoint was the proportion of heavy‐drinking days (PHDD) over the 24‐week observation period. Secondary endpoints included changes in PHDD and total drinking volume within earlier time windows (Weeks 1–4, 5–8 and 9–12). Heavy drinking was defined as ≥ 5 standard drinks/day for men or ≥ 4 for women, with 1 standard drink equivalent to 10 g of ethanol.

For the prespecified mechanistic lipidomics substudy, plasma sampling was performed at Week 12, which was selected a priori as a time point within the sustained‐release window of the naltrexone implant, following resolution of the early post‐detoxification metabolic fluctuation period [29], and sufficiently early to allow prospective association analyses with drinking outcomes during the subsequent 12‐week observation window (Weeks 13–24). Among NI participants with available Week‐12 plasma samples and successful lipidomic profiling, treatment response was classified using PHDD over the 24‐week observation period; descriptive baseline characteristics of included and non‐included NI participants are shown in Table S1d. Participants with PHDD ≤ 10% were defined a priori as RN (*n* = 18), whereas those with PHDD > 10% were classified as nonresponders (NR, *n* = 10). The 10% threshold was prespecified as a stringent criterion for infrequent heavy drinking, informed by earlier alcohol treatment studies in which ≤ 10% heavy drinking days was used as part of a favourable or moderate drinking outcome definition [30]. From the PL arm, 10 PL participants were randomly selected from those with available Week‐12 plasma samples and successful profiling. Healthy controls (HC) consisted of 10 community‐recruited male participants frequency‐matched to the lipidomics substudy sample on sex, age and body mass index (BMI). This responder‐enriched design was prespecified as an exploratory mechanistic strategy to enhance biological signal detection under finite sample and assay constraints, rather than to estimate the average lipidomic effect across all naltrexone‐treated participants.

### 2.2 Lipidomics Data Acquisition, Processing and Normalisation

For the lipidomics substudy described above, fasting venous blood was collected in the morning into EDTA tubes. Blood samples were allowed to stand at room temperature for 20 min and were then centrifuged at 3000 rpm for 10 min at 4°C. Plasma supernatant (500 μL) was aliquoted into 2 mL cryovials and stored at −80°C until lipidomic profiling.

Plasma lipids were extracted using a standard methyl tert‐butyl ether (MTBE) protocol. Untargeted LC–MS lipidomics analysis was performed on a Q Exactive HF‐X Orbitrap with a C30 column, acquiring full‐scan MS (m/z 200–2000) and data‐dependent MS/MS in ESI± modes. Pooled QC samples were injected every 10 samples for quality control [31].

Raw files were processed in LipidSearch for peak detection, alignment and MS/MS‐based identification. The resulting feature table was filtered to retain features detected in ≥ 80% of samples in at least one study group and to remove features with pooled‐QC RSD > 30%. Ratios and proportions were computed using a pairwise‐complete strategy; cases with missing or non‐positive denominators were excluded from that comparison.

Probabilistic quotient normalisation (PQN) was employed as the primary normalisation strategy to correct for sample dilution differences [32, 33, 34], using the HC median spectrum as the reference $r_{i}$. For each sample j, a dilution factor $s_{j}=\text{median}\left(x_{\mathrm{ij}}/r_{i}\right)$ was computed, and PQN‐scaled intensities were $y_{\mathrm{ij}}=x_{\mathrm{ij}}/s_{j}.$ PQN‐scaled data were then log10‐transformed for statistical tests requiring approximate normality. For exploratory multivariate visualisation, principal component analysis (PCA) was performed on the log10‐transformed data after Pareto scaling to visualise global structure and assess potential outliers and was used for visualisation only rather than hypothesis testing. All downstream statistical analyses and statistical graphics were performed in R (version 4.4.1).

### 2.3 Class‐Level and Pathway Statistical Analysis

Species‐level intensities were aggregated (summed) to obtain a sample × class matrix by first back‐transforming log10(PQN) values to the linear scale within each sample, summing across species within the same class, and then re‐transforming to log10 for analysis. The prespecified classes were PC, PE, phosphatidylethanol (PEt), *N*, *N*‐dimethylphosphatidylethanolamine (DMPE), lysophosphatidylcholine (LPC), sphingomyelin (SM), ceramide (Cer) and phosphatidylinositol (PI), together with the endocannabinoid anandamide (AEA; arachidonoylethanolamide). Statistical comparisons primarily focused on two prespecified contrasts: RN versus PL and PL versus HC. To further characterise response heterogeneity, exploratory comparisons were additionally performed for NR versus PL. For each class, group differences were assessed on the log10 class totals using Welch's *t*‐test. For each contrast, effect sizes were reported as Δlog10 (group difference on the log10 scale) and fold‐changes were derived as FC = 10^Δlog10. Primary reporting focused on RN versus PL, whereas NR versus PL was analysed exploratorily using the same framework. *p*‐values across classes were adjusted using the Benjamini–Hochberg (BH) procedure to control the false discovery rate (FDR), yielding *q*‐values; the main reporting threshold was *q* < 0.05 [35].

### 2.4 Quantifying Molecular Recovery

A central goal of the analysis was to quantify molecular ‘recovery’, defined as the treatment‐driven reversal of disease‐associated lipid changes back toward a healthy state. To visualise and quantify this at the individual species level, we constructed a two‐contrast recovery scatterplot.

The *y*‐axis represented the ‘disease contrast’ (log2FC PL vs. HC), and the *x*‐axis represented the ‘treatment contrast’ (log2FC RN vs. PL), computed as log2 fold‐changes from PQN‐scaled linear intensities using group medians:

$$y_{i}=\log_{2}\left(\frac{\text{median}\left(X_{i,\mathrm{PL}}\right)}{\text{median}\left(X_{i,\mathrm{HC}}\right)}\right),x_{i}=\log_{2}\left(\frac{\text{median}\left(X_{i,\mathrm{RN}}\right)}{\text{median}\left(X_{i,\mathrm{PL}}\right)}\right).$$

Here, *i* is the index lipid species (features), *s* is the index samples, and $X_{i,s}$ denotes the PQN‐scaled intensity on the linear scale for lipid *i* in sample *s*.

Recovery was specifically defined as lipids residing in the upper‐left quadrant (*y* > 0 and *x* < 0). This quadrant represents species that were elevated in the disease state (PL vs. HC) and subsequently reversed (decreased) by the naltrexone treatment in responders (RN vs. PL). A continuous recovery strength score ($S_{i}$) was calculated ($S_{i}=\max\left(0,-x_{i}\right)+\max\left(0,y_{i}\right)$) to identify the lipids with the strongest recovery. Point opacity in the plot reflected the statistical significance (Welch's *p*‐value) of the treatment contrast.

### 2.5 Analysis of Lipid Ratios and Fractions

Additional analyses were performed on PQN‐normalised data. We calculated ratios between major lipid class total abundances (e.g.,$\mathrm{PC}/{\mathrm{PE}}_{\text{total}}$, DMPE/${\mathrm{PE}}_{\text{total}}$) and intraclass fractions representing the proportion of specific fatty acyl species within their parent class (n‐3 PUFAs and AA within PC and PE pools, respectively). Class totals were computed by back‐transforming log10(PQN) values to the linear scale within each sample and summing across species within the same class. Ratios were log10‐transformed, and fractions (constrained to [0, 1]) were logit‐transformed prior to statistical testing.

The primary contrasts of interest were RN versus PL and PL versus HC, assessed using Welch's *t*‐test on the transformed indices. Within each contrast, *p*‐values were adjusted across the six indices using BH‐FDR. For display, effect sizes for class ratios are reported as mean differences on the log10 scale (Δlog10), and those for intraclass fractions are reported as mean differences on the logit scale; intraclass fractions are additionally reported as percentage‐point differences for visual reference in figures.

To further characterise response specificity, exploratory comparisons were performed for NR versus PL and RN versus NR at Week 12 using the same Welch's *t*‐test framework, with BH‐FDR adjustment applied across the six indices within each comparison. Results are presented as between‐group mean differences with 95% confidence intervals in a forest plot format.

### 2.6 Correlation of Week‐12 Lipid Indices With Heavy‐Drinking Outcomes

We examined associations between prespecified Week‐12 lipid indices and drinking outcomes in the RN and PL groups (*n* = 28). Indices were PC/PE (log10), DMPE/PE (log10), n‐3 in PC (logit), n‐3 in PE (logit), AA in PC (logit) and AA in PE (logit); fractions were logit‐transformed and ratios log10‐transformed prior to testing. Two outcomes were analysed: PHDD (Weeks 13–24), defined as the PHDD during study Weeks 13–24, and PHDD (overall) as provided in the trial dataset. Associations were assessed using two‐sided Spearman rank correlations on complete cases. Within each time window, *p*‐values were adjusted across the six indices using BH‐FDR.

## 3 Results

### 3.1 Clinical Efficacy and Patient Cohorts

Seventy participants with AUD were randomised. The prespecified primary endpoint—PHDD over Weeks 1–24—favoured naltrexone (median 1.49% vs. 13.39%; Hodges–Lehmann Δ − 5.95 percentage points, 95% CI − 23.21 to 0.00; *p* = 0.042). Late‐window secondary analyses (Weeks 21–24) were directionally consistent (RR 0.50, 95% CI 0.26–0.97). A concise summary of the primary and key secondary outcomes is provided in Table 1; full model–based estimates (GEE, exchangeable, robust SE) are shown in Table S2. Baseline demographics, clinical characteristics, laboratory parameters and the Week‐12 lipidomics subset comparison (RN *n* = 18, NR *n* = 10, PL n = 10, HC n = 10) are summarised in Table S1. Baseline profiles were broadly comparable across the clinical subgroups, whereas HCs were frequency‐matched on sex, age and BMI.

**TABLE 1:** Primary and key secondary outcomes.

Outcome	NI *n*	PL n	NI value	PL value	Effect	95% CI	*p*
PHDD, Weeks 1–24	46	20	1.49 (0.00–10.71)	13.39 (1.79–48.66)	HL Δ = −5.95 pp	−23.21 to 0.00	0.042
Heavy drinking days, Weeks 21–24	46	23	—	—	RR = 0.50	0.26–0.97	0.041
Change in heavy drinking days, Weeks 21–24	46	23	—	—	Δ = −6.24 days	−11.75 to −0.73	0.026

*Note:* PHDD, percentage of heavy‐drinking days (≥ 5 standard drinks/day for men or ≥ 4 for women; 1 standard drink = 10 g ethanol). Values are median (IQR) for the PHDD row. The primary comparison used the Mann–Whitney *U* test with Hodges–Lehmann (HL) median difference (percentage points). Secondary outcomes are generalised estimating equation (GEE) contrasts for Weeks 21–24, reported as rate ratio (RR) or absolute difference in heavy‐drinking days. Effects are coded as NI − PL, with negative values favouring NI. *n* indicates the number of participants with non‐missing data. Full longitudinal estimates are shown in Table S2.

### 3.2 Class‐Level Lipidomic Signature of Naltrexone Response

**FIGURE 1:**
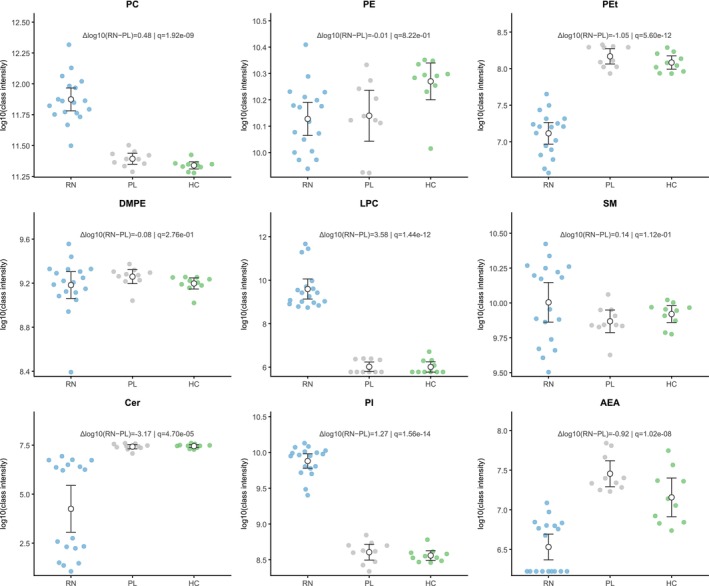
Class‐level lipid differences at Week 12. Beeswarm plots for nine classes—PC, PE, PEt, DMPE, SM, Cer, LPC, PI, and AEA—across responders to naltrexone (RN, *n* = 18), placebo (PL, *n* = 10), and healthy controls (HC, *n* = 10). Points are individual samples; white circles with whiskers show group mean ± 95% CI. Values are log10 of within‐class summed PQN‐normalised intensities. Annotations report Δlog10(RN − PL) and BH‐FDR q from two‐sided Welch's *t*‐tests; *q* < 0.05 was considered significant.

Exploratory PCA suggested separation across groups (Figure S1). Across eight prespecified lipid classes (PC, PE, PEt, DMPE, LPC, SM, Cer and PI) plus AEA, RN versus PL showed robust increases in total PC (Δlog10 = 0.481, *q* = 1.92 × 10^−9^; Hedges' g = 3.01, 95% CI 1.89–4.14; 105 species) and PI (Δlog10 = 1.274, *q* = 1.56 × 10^−14^; g = 6.61, 95% CI 4.65–8.56; 18 species). In contrast, PEt, Cer and AEA were lower in RN (all *q* ≤ 4.7 × 10^−5^; g range −1.58 to −4.00), whereas PE, DMPE and SM showed no significant differences (q > 0.10; |g| ≤ 0.55). These class‐level lipid differences are summarised in Figure 1. Full statistics are provided in Table S3.

Exploratory cross‐sectional comparisons between NR and PL at Week 12 showed that several class‐level differences were not restricted to responders. Specifically, NR also differed from PL in total PI, PEt, LPC, AEA, PC and Cer after BH‐FDR correction, whereas PE, SM and DMPE were not significantly different (Table S4a).

### 3.3 Per‐Lipid Recovery Map

**FIGURE 2:**
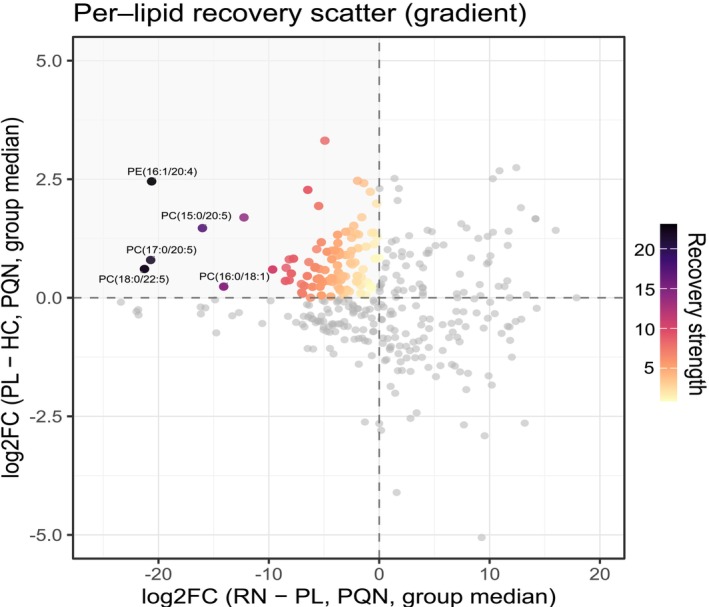
Per‐lipid recovery map (RN–PL vs PL–HC). Each point is a lipid species. The *y*‐axis shows the disease contrast (group‐median log2FC, PL vs. HC) and the *x*‐axis the treatment contrast (group‐median log2FC, RN vs. PL), computed from PQN‐normalised data. Points in the upper left quadrant (*y* > 0, *x* < 0) indicate ‘recovery’. Point colour encodes a composite recovery strength (greater leftward treatment shift and upward disease shift = stronger recovery). Point opacity encodes statistical weight as −log10(p for RN–PL by Welch's t on the log10(PQN) scale), capped at 10; higher opacity means smaller p. Dashed lines mark zero. Representative top‐ranked recoveries are labelled; full per‐species statistics are provided in the source data.

The per‐lipid recovery map (Figure 2) showed a clear concentration of features in the recovery quadrant, indicating broad reversal of disease‐associated elevations in responders. Among the highest‐ranking recoveries were PE (16:1/20:4), PC (15:0/20:5), PC (17:0/20:5), PC (18:0/22:5) and PC (16:0/18:1); full per‐species statistics are provided in Table S5. To determine whether these species‐level reversals converged on broader membrane‐remodelling patterns, we next examined prespecified axis‐level indices summarising headgroup balance and acyl‐chain composition.

### 3.4 Axis‐Level Shifts in Membrane Phospholipids

**FIGURE 3:**
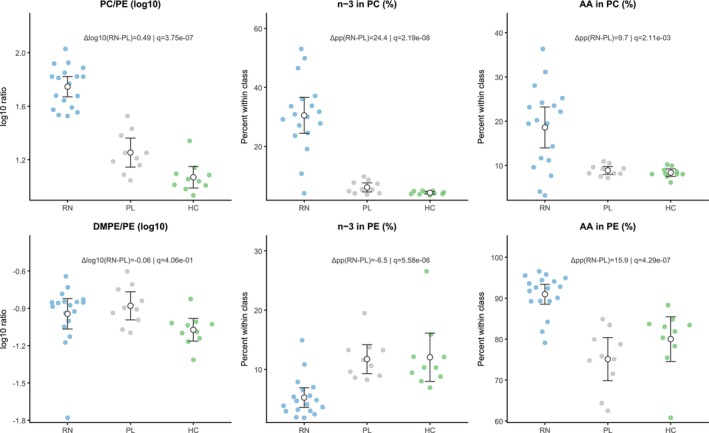
Axis‐level indices of membrane phospholipids. Beeswarm plots for RN (blue), PL (grey) and HC (green); points are individual samples, and white circles with whiskers show group mean ± 95% CI. Ratios (PC/PE, DMPE/PE) were computed by back‐transforming log10(PQN) to linear, summing within class, forming the ratio, then taking log10. Fractions are within‐class percentages (n‐3 = EPA + DHA = 20:5, 22:6; AA = 20:4) and were tested on the logit scale. Annotations report Δlog10(RN−PL) for ratios or Δ percentage points for fractions and the BH‐FDR q (two‐sided Welch's *t*; FDR across the six prespecified indices).

The recovery map was led by PC/PE species, motivating axis‐level summaries on PEMT and Lands. At Week 12, RN versus PL showed coordinated differences across six prespecified indices (Figure 3). On the PEMT axis, PC/PE was higher in RN than PL (*q* < 0.05; Hedges' g = 3.13, 95% CI 1.98–4.29), whereas DMPE/PE did not meet the FDR threshold. On the Lands axis, the n‐3 fraction in PC was higher (g = 2.37, 95% CI 1.36–3.38) and the n‐3 fraction in PE was lower (g = −1.88, 95% CI ‐2.80 to −0.95) in RN; AA fractions were higher in both PC (g = 1.25, 95% CI 0.40–2.09) and PE (g = 2.62, 95% CI 1.57–3.67) (both *q* < 0.05). Full effect sizes and statistics are provided in Table S6b.

PL versus HC showed selective separation across the six prespecified membrane indices, with PL exhibiting higher values than HC for n‐3 in PC, PC/PE and DMPE/PE after BH‐FDR correction, whereas n‐3 in PE, AA in PC and AA in PE were not significantly different (Table S6c). Exploratory RN vs. HC comparisons further showed that several Lands‐cycle–related indices remained different in responders relative to HCs (Table S6d).

Exploratory NR analyses refined this axis‐level interpretation. Relative to PL, NR showed significant differences in n‐3 in PC and n‐3 in PE after BH‐FDR correction, whereas PC/PE, DMPE/PE, AA in PC and AA in PE were not significant (Table S4b). Direct RN versus NR comparison did not yield BH‐FDR‐significant differences for any of the six indices, although PC/PE showed the clearest between‐subgroup separation trend (difference = 0.315, *p* = 0.015, *q* = 0.093; Table S4c). These subgroup comparisons are visualised in the forest plot shown in Figure S2. Taken together, the findings suggest partial overlap between RN and NR in Lands‐cycle‐related remodelling, with more pronounced separation on the headgroup‐balance axis.

### 3.5 Membrane Flux Readouts Correlate With Heavy‐Drinking Burden

**FIGURE 4:**
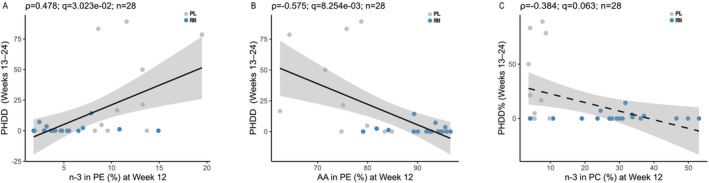
Correlations between Week‐12 lipid indices and subsequent heavy‐drinking burden (Weeks 13–24). (A) n‐3 in PE (%) versus PHDD (Weeks 13–24). (B) AA in PE (%) versus PHDD (Weeks 13–24). (C) n‐3 in PC (%) versus PHDD (Weeks 13–24). Each dot is one participant (RN = blue, PL = grey). Lines show least‐squares fits with 95% CI shading; the dashed line in panel C denotes a trend.

In RN + PL (*n* = 28), two Week‐12 readouts showed significant prospective associations with PHDD during Weeks 13–24: higher n‐3 in PE was associated with higher PHDD (Spearman's *ρ* = 0.478, *q* = 3.02 × 10^−2^), and higher AA in PE was associated with lower PHDD (*ρ* = −0.575, *q* = 8.25 × 10^−3^) (Figure 4A,B). PC/PE, n‐3 in PC and AA in PC showed directionally consistent associations but did not survive BH‐FDR correction (*ρ* = −0.370 to −0.392, *q* = 6.31 × 10^−2^), whereas DMPE/PE showed no association (*ρ* = 0.135, *q* = 0.492). The overall PHDD window showed consistent associations for n‐3 in PE (*ρ* = 0.585, *q* = 3.22 × 10^−3^) and AA in PE (*ρ* = −0.622, *q* = 2.44 × 10^−3^) and additionally revealed a significant inverse association for n‐3 in PC (*ρ* = −0.470, *q* = 2.32 × 10^−2^). Full statistics (*n*, *ρ*, *p*, *q*) are provided in Table S7.

## 4 Discussion

In this randomised, double‐blind, PL‐controlled trial of a long‐acting naltrexone implant, we found that, within a prespecified Week‐12 mechanistic lipidomics substudy, individuals meeting a prespecified responder criterion based on PHDD over the 24‐week observation period exhibited a coherent, membrane‐centric plasma lipidomic phenotype. Across class‐, species‐ and pathway‐level analyses, responders differed from PL‐treated participants in a coordinated manner, and several axis‐level indices measured at Week 12 were associated prospectively with later heavy‐drinking burden.

At the class level, the responder group showed prominent shifts in major membrane lipids, most notably higher total PC and PI and lower PEt, ceramide and anandamide relative to PL. These class‐level patterns provide a biologically interpretable backdrop for the more granular species‐ and axis‐level findings. Ceramide reductions are directionally consistent with a lower sphingolipid‐associated lipotoxic/inflammatory tone described across metabolic stress states and AUD‐related physiology [36, 37, 38]. Likewise, the decrease in plasma AEA in responders aligns with the broader literature implicating endocannabinoid tone in alcohol reinforcement, stress responsivity and relapse vulnerability [39, 40, 41]. In this context, lower AEA may reflect a responder‐associated shift in reward‐ and stress‐related lipid signalling during treatment. Because AEA was measured here as a single species on an untargeted platform, targeted quantification of AEA and related endocannabinoids will be useful for clarifying its temporal relationship to drinking behaviour.

At the species level, our ‘recovery map’ (Figure 2) leveraged two orthogonal contrasts (PL vs. HC and RN vs. PL) to visualise directional reversal. The concentration of features in the recovery quadrant indicates that many disease‐associated elevations observed in PL‐treated participants moved toward the healthy‐control direction among responders. At the same time, the map also showed that a subset of species in responders occupied values outside the healthy‐control central tendency, consistent with a treatment‐associated remodelling state rather than a strict return to a baseline ‘normal’. This distinction matters for interpretation: A clinically beneficial state may reflect an adaptive membrane configuration achieved during treatment and abstinence rather than a simple reversal of all AUD‐linked deviations.

**FIGURE 5:**
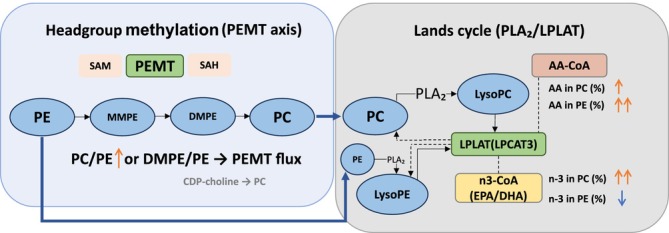
PEMT axis and Lands cycle. Proposed integration of the lipidomic findings in responders versus placebo, showing higher PC/PE on the headgroup axis and coordinated AA enrichment with reciprocal n‐3 redistribution across phospholipid pools on the Lands‐cycle axis.

These multilayer patterns converge mechanistically on two established axes of membrane remodelling highlighted in Figure 5: headgroup balance (PC/PE) and Lands‐cycle acyl‐chain editing.

On the headgroup axis, responders showed a higher PC/PE ratio. PC/PE is a biophysical determinant of bilayer packing, curvature stress and organelle homeostasis [24], and prior work has linked low PC/PE to membrane fragility and hepatometabolic injury. Notably, PC/PE followed a graded pattern across groups—HC < PL < RN—indicating that elevation begins during the post‐detoxification period and is further amplified in responders, rather than representing a simple normalisation toward healthy levels. PC/PE in plasma integrates multiple sources, including PEMT flux, the Kennedy pathway, lipoprotein assembly/export and dietary inputs. Accordingly, we interpret elevated PC/PE as reflecting a treatment‐associated shift toward a more PC‐enriched membrane state. The absence of a significant rise in DMPE/PE argues against simple methylation intermediate accumulation, but does not exclude compartment‐specific PE turnover.

On the acyl‐chain axis, responders showed coordinated changes in phospholipid acyl‐chain composition, including AA enrichment in both PC and PE and a cross‐headgroup redistribution of n‐3 PUFAs (higher n‐3 in PC and lower n‐3 in PE). This pattern is compatible with active reacylation and selective remodelling within the Lands cycle, potentially involving PLA_2_–lysophospholipid turnover and preferential incorporation of specific PUFA‐CoA donors by lysophospholipid acyltransferases [42]. LPCAT3 and related enzymes have been shown to shape AA‐enriched phospholipid pools in hepatocytes and enterocytes [22], providing a biologically plausible but tissue‐unresolved route for this signal. From an addiction‐biology perspective, the key point is not only the identity of the enzyme but the implication of membrane composition: Shifts in headgroup balance and PUFA distribution can, in principle, influence receptor microenvironments, GPCR signalling efficiency, vesicular dynamics and neuroimmune tone—processes that are repeatedly implicated in craving and relapse risk [43].

Axis‐level comparisons suggested two patterns. PC/PE, DMPE/PE and n‐3 in PC were already elevated in PL relative to HC, indicating membrane shifts present independent of treatment response. By contrast, n‐3 in PE, AA in PC and AA in PE were more specific to RN, identifying these Lands‐cycle indices as more treatment‐associated. Exploratory subgroup comparisons refined this view: NR differed mainly in n‐3‐related indices, whereas RN showed broader shifts spanning both axes. Although RN versus NR did not survive BH‐FDR correction, PC/PE showed the clearest separation trend, supporting its interpretation as a responder‐enriched headgroup‐balance signal.

This distinction also helps integrate the prospective correlation results. Week‐12 Lands‐cycle readouts—especially n‐3 in PE and AA in PE, and to a lesser extent n‐3 in PC—were associated with later heavy‐drinking burden, suggesting that these indices may capture the degree or quality of membrane adaptation that remains relevant to subsequent clinical course rather than responder status alone. By contrast, PC/PE showed the strongest enrichment in RN and the clearest trend for RN versus NR separation, supporting the view that headgroup balance may better mark the responder‐associated configuration itself. In this framework, the two axes appear complementary rather than redundant: partial Lands‐cycle indices may track outcome‐linked membrane remodelling, whereas PC/PE may better reflect responder‐enriched membrane reorganisation.

These responder‐specific patterns need to be interpreted against the background of abstinence‐related lipid change, which has its own characteristic signature distinct from treatment‐associated remodelling. Even without anti‐relapse pharmacotherapy, alcohol withdrawal can be accompanied by selective shifts in phospholipids and related lipid‐metabolic readouts. In a recent metabolomic study of 96 hospitalised patients with severe AUD, a 3‐week detoxification period was associated with decreases in several phospholipid species that were elevated at baseline, including selected PC‐ and LPC‐related signals, whereas some baseline‐reduced odd‐chain LPC species increased but did not fully return to control levels [28]. Not all lipids behaved in the same way, however, indicating that abstinence‐related lipid changes are class‐specific rather than uniform. Earlier studies similarly showed selective normalisation of specific lipid markers—including oxidative phospholipid species and lipoprotein‐related abnormalities—following short‐term abstinence, but without uniform restoration across lipid classes [27, 44].

Against this background, the present findings support an association with a more coherent membrane‐remodelling pattern during naltrexone treatment, beyond what would be expected from a nonspecific lipid shift alone. In our dataset, PEt likely captured one dimension linked more closely to ethanol exposure, whereas the coordinated alterations in PC/PE balance and PUFA distribution across PC and PE point to a broader reorganisation of membrane lipid composition. This interpretation is also consistent with the exploratory NR analyses: Lipid perturbations were not absent in NR, but the responder group showed a more coherent, membrane‐centred pattern across class‐ and axis‐level readouts. Taken together, these considerations suggest that abstinence‐related lipid changes likely form part of the biological context in which our signals emerged, while the more integrated dual‐axis pattern identified here appears to be more closely aligned with clinical response.

This study has several strengths: a randomised, double‐blind design; prespecified clinical endpoints; and convergent lipidomic signals spanning class totals, species‐level directional mapping, and prespecified pathway indices, with prospective linkage to clinically meaningful drinking outcomes.

Limitations should temper inference. The mechanistic lipidomics analysis was performed in a responder‐enriched subset at a single on‐treatment time point, which improves sensitivity to detect biological signals but complicates causal attribution and may amplify differences related to drinking reduction itself. Blood samples for Week‐24 lipidomic analysis were not collected, limiting longitudinal interpretation of the on‐treatment membrane phenotype. Furthermore, the cross‐sectional PL versus HC comparison does not allow causal attribution of observed differences to AUD pathology versus recovery‐related or lifestyle factors. Although exploratory subgroup comparisons suggested partial overlap between NR and RN on several membrane indices, these analyses were based on a small sample and did not yield BH‐FDR‐significant RN versus NR separation, and therefore should be interpreted cautiously. The subset included only males, limiting generalisability. In addition, HCs were matched on sex, age and BMI, but not on smoking status; therefore, residual confounding by smoking cannot be excluded. Plasma is a peripheral proxy that cannot localise changes to brain versus liver versus gut, and untargeted annotations require targeted validation for key species, including AEA. Finally, replication in independent cohorts will be necessary before clinical translation.

In summary, response to long‐acting naltrexone in AUD was associated with a dual‐axis membrane remodelling phenotype—PC/PE headgroup balance and Lands‐cycle–linked PUFA partitioning—measurable in plasma and related to subsequent heavy‐drinking burden. These membrane‐centric indices provide a biologically grounded framework for future work integrating longitudinal lipidomics with targeted enzymatic and endocannabinoid assays, and for testing whether modulating phospholipid remodelling can enhance treatment outcomes.

## Author Contributions

Liyao Huang: conceptualisation, methodology, formal analysis, data curation, visualisation, writing – original draft, writing – review and editing. Bojie Zhou: Conceptualisation, investigation, resources, methodology, project administration, writing – review and editing. Qinling Ou: data curation, formal analysis, methodology, validation, writing – review and editing. Jubo Yin: project administration, data curation, resources, writing – review and editing. Xiwen Tian: formal analysis, methodology, validation, writing – review and editing. Xuhui Zhou: conceptualisation, funding acquisition, supervision, project administration, investigation, validation, writing – review and editing.

## Funding

This study was supported by the Key Project of Natural Science Foundation of Hunan Province (No. 2026JJ30086), Hunan Provincial Clinical Research Center for Addiction Disorder (No. 2023SK4055), Hunan Provincial Health High‐Level Talent Scientific Research Project (No. R2023178), National Cultivation Project of Key Clinical Specialty (Addiction medicine), Hunan Province clinical key specialty (Addiction medicine), and Key Clinical Specialty Construction Project of the Hunan Health Commission (Improvement of Diagnosis and Treatment Ability of Severe Psychiatric Diseases in Hunan Province).

## Ethics Statement

The study was approved by the Ethics Committee/Institutional Review Board of Hunan Second People's Hospital (approval no. Y2022022) and conducted in accordance with the Declaration of Helsinki. All participants provided written informed consent prior to participation.

## Conflicts of Interest

The authors declare no conflicts of interest.

## Supporting information


**DATA S1:** Supporting Information.

**Figure S1:** Principal component analysis (PCA) of PQN‐normalised lipidomic profiles. Scores plot shows PC1 vs. PC2 for RN (blue), PL (grey) and HC (green) with 95% confidence ellipses. Data were log10‐transformed (PQN‐scaled) and Pareto‐scaled prior to PCA.

**Figure S2:** Exploratory between‐group differences in membrane‐related lipid indices at Week 12. Mean differences (95% CI) for RN versus PL (circles) and NR versus PL (triangles) across six prespecified axis‐level indices. Class ratios (PC/PE, DMPE/PE) are shown on the log10 scale and intraclass fractions (n‐3 in PC, AA in PC, n‐3 in PE, AA in PE) on the logit scale. *p* values were BH‐FDR adjusted within each comparison. NR, naltrexone nonresponders; PL, placebo; RN, naltrexone responders.

**Table S1a:** Baseline demographics and clinical characteristics in the randomised cohort.
**Table S1b:** Baseline laboratory parameters in the randomised cohort.
**Table S1c:** Baseline characteristics across the four groups.
**Table S1d:** Descriptive baseline characteristics of NI participants included versus not included in the lipidomics subset.

**Table S2:** Primary and secondary outcomes (GEE, exchangeable, robust SE).

**Table S3:** Supporting Information.

**Table S4:** Exploratory analyses in naltrexone nonresponders (NR).

**Table S5:** Supporting Information.

**Table S6:** Axis‐level statistics for primary contrasts at Week 12.

**Table S7:** Supporting Information.

## Data Availability

Raw LC–MS files will be deposited in MetaboLights and made publicly available upon publication. Custom R scripts used in this study are available from the corresponding author upon reasonable request.
