# Supplementary material for: A Membrane‐Centric Plasma Lipidomic Signature of Response to Long‐Acting Naltrexone in Alcohol Use Disorder
Source: Addict Biol. 2026 May 12;31(5):e70165. doi: 10.1111/adb.70165 (PMC13167251; doi:10.1111/adb.70165)
Supplement: Supplementary file 1 — DATA S1: Supporting Information. [file ADB-31-e70165-s010.docx]

***Supplementary Methods***

***Eligibility Criteria***

Inclusion criteria

Participants were eligible for enrollment only if all of the following criteria were met:

1. They voluntarily agreed to participate, provided written informed consent, and were willing and able to comply with all study procedures.
2. They were aged 18 years or older at the time of informed consent.
3. They met DSM-5 criteria for moderate-to-severe alcohol use disorder, defined as the presence of at least four diagnostic criteria.
4. They had completed detoxification treatment and had remained free of clinically significant alcohol withdrawal symptoms for at least 7 days before randomization and implant administration, with a Clinical Institute Withdrawal Assessment for Alcohol, revised (CIWA-Ar) score <7.
5. They were able to provide Timeline Follow-Back (TLFB) drinking data for the 2 weeks preceding detoxification and/or screening.
6. They had engaged in heavy drinking at least twice weekly during the 4 weeks preceding detoxification and/or screening.
7. Participants of childbearing potential agreed to use effective contraception throughout the study period. Women were considered not of childbearing potential if they were postmenopausal, defined as at least 12 consecutive months of amenorrhea without an alternative medical cause, or had undergone hysterectomy or bilateral oophorectomy.

Exclusion criteria

Participants were excluded if any of the following criteria were present:

1. Any condition that, in the investigator’s judgment, made participation not in the participant’s best interest or rendered study participation unsafe.
2. Pregnancy, lactation, a positive pregnancy test at screening, or plans to become pregnant during the study period.
3. Clinically significant hepatic dysfunction, including AST or ALT >2 × the upper limit of normal, liver failure, or other hepatobiliary abnormalities that could interfere with evaluation of study efficacy or safety. Liver failure included, but was not limited to, ascites, prolonged prothrombin time, international normalized ratio (INR) ≥1.7, or esophageal varices.
4. Clinically uncontrolled active infection, including active hepatitis B infection (HBsAg-positive with HBV DNA >1000 IU/mL) or active hepatitis C infection (anti-HCV positive with detectable HCV RNA).
5. A history of congenital bleeding disorders, any active clinically significant bleeding, platelet dysfunction, prothrombin time more than 3 seconds above the upper limit of normal, or platelet count <50 × 10^9/L.
6. A history of severe pancreatitis or severe delirium tremens.
7. Any severe or uncontrolled systemic disease or major psychiatric disorder that, in the investigator’s judgment, could compromise informed consent, participant safety, protocol adherence, or interpretation of study outcomes.
8. Expected hospitalization or surgery during the study period, including planned elective surgery or hospitalization that could not be postponed.
9. A current diagnosis of a non-alcohol substance use disorder within 1 year before randomization, according to DSM-5 criteria, including but not limited to benzodiazepine-, amphetamine-, opioid-, or cocaine-related disorders.
10. Use of anti-relapse medication for alcohol use disorder, such as naltrexone, or receipt of structured psychosocial treatment within 30 days before randomization and implant administration.
11. Current opioid therapy, opioid use within 7 days before randomization and implant administration, anticipated need for opioid treatment during the study period, a positive urine drug screen for opioids, cannabis, amphetamines, or other relevant substances on the day of randomization, or a positive naloxone challenge test.
12. Known hypersensitivity to the investigational product, its excipients (including polylactic acid or magnesium stearate), or local anesthetics.
13. Current participation in another investigational drug or device study, or use of any investigational drug or device within 30 days before randomization and implant administration.
14. Skin infection at the implantation site or a systemic skin disorder that could interfere with evaluation of treatment efficacy or safety.
15. Clinical or laboratory evidence of human immunodeficiency virus infection or syphilis.
