# Supplementary figures and images for: A Membrane‐Centric Plasma Lipidomic Signature of Response to Long‐Acting Naltrexone in Alcohol Use Disorder
Source: Addict Biol. 2026 May 12;31(5):e70165. doi: 10.1111/adb.70165 (PMC13167251; doi:10.1111/adb.70165)

PCA on log10(PQN) (Pareto-scaled per feature)

PC1 (63.5%)

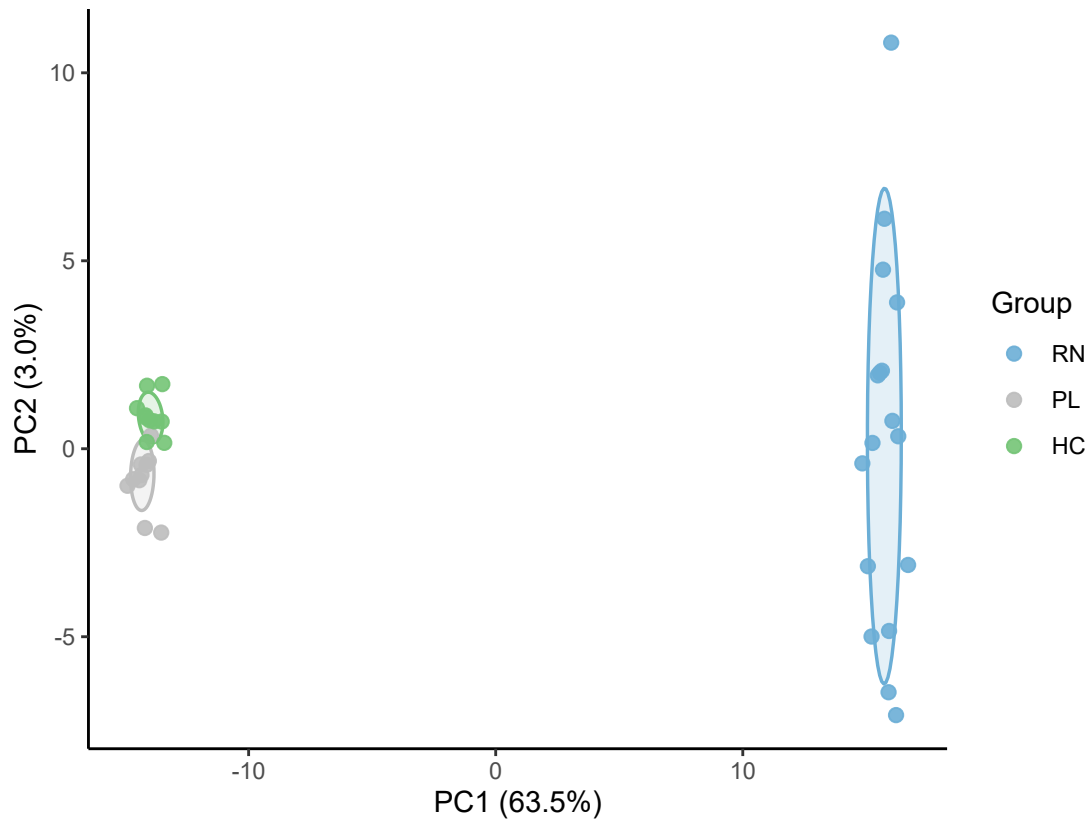

Supplement: Supplementary file 2 — Figure S1: Principal component analysis (PCA) of PQN‐normalised lipidomic profiles. Scores plot shows PC1 vs. PC2 for RN (blue), PL (grey) and HC (green) with 95% confidence ellipses. Data were log10‐transformed (PQN‐scaled) and Pareto‐scaled prior to PCA. [file ADB-31-e70165-s004.pdf]

## Membrane axis indices at Week 12: responders vs non-responders

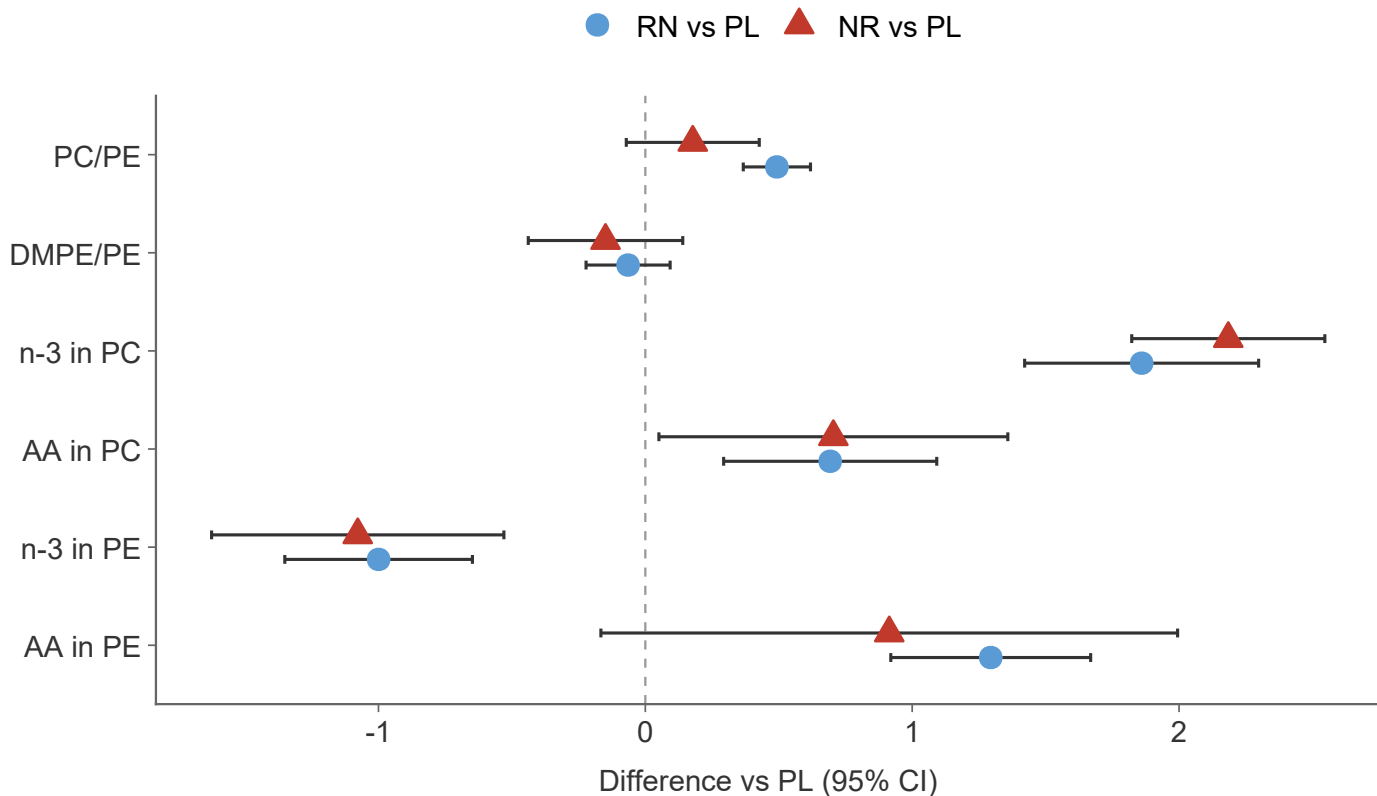

Supplement: Supplementary file 3 — Figure S2: Exploratory between‐group differences in membrane‐related lipid indices at Week 12. Mean differences (95% CI) for RN versus PL (circles) and NR versus PL (triangles) across six prespecified axis‐level indices. Class ratios (PC/PE, DMPE/PE) are shown on the log10 scale and intraclass fractions (n‐3 in PC, AA in PC, n‐3 in PE, AA in PE) on the logit scale. p values were BH‐FDR adjusted within each comparison. NR, naltrexone nonresponders; PL, placebo; RN, naltrexone responders. [file ADB-31-e70165-s003.pdf]
