# Supplementary material for: A Membrane‐Centric Plasma Lipidomic Signature of Response to Long‐Acting Naltrexone in Alcohol Use Disorder
Source: Addict Biol. 2026 May 12;31(5):e70165. doi: 10.1111/adb.70165 (PMC13167251; doi:10.1111/adb.70165)
Supplement: Supplementary file 4 — Table S1a: Baseline demographics and clinical characteristics in the randomised cohort. Table S1b: Baseline laboratory parameters in the randomised cohort. Table S1c: Baseline characteristics across the four groups. Table S1d: Descriptive baseline characteristics of NI participants included versus not included in the lipidomics subset. [file ADB-31-e70165-s001.docx]

**Table S1a. Baseline demographics and clinical characteristics in the randomized cohort**

| Characteristic | Naltrexone implant (NI, n=46) | Placebo (n=23) | Test statistic |  | *P value* |
| --- | --- | --- | --- | --- | --- |
| Gender |  |  | — |  | 1 |
| Male | 45(97.8) | 22(95.7) |  |  |  |
| Female | 1(2.2) | 1(4.3) |  |  |  |
| Age (years) | 42±9.80 | 43±10.14 | 0.395 |  | 0.694 |
| Height (cm) | 170±6.21 | 170±5.53 | 0.412 |  | 0.682 |
| Weight (kg) | 65.5(57.0, 70.5) | 65.0(55.0, 75.0) | -0.312 |  | 0.755 |
| BMI (kg/m²) | 22.6(20.5, 23.8) | 21.4(20.1, 23.9) | -0.764 |  | 0.445 |
| Previous Inpatient Detoxification Treatment |  |  | 0.338 |  | 0.561 |
| Yes | 35(76.1) | 16(69.6) |  |  |  |
| No | 11(23.9) | 7(30.4) |  |  |  |
| Years of Education | 12(9, 15) | 12(12, 16) | -0.505 |  | 0.614 |
| Annual Income ≥ 100,000 CNY |  |  | 1.725 |  | 0.189 |
| Yes | 11(23.9) | 9(39.1) |  |  |  |
| No | 35(76.1) | 14(60.9) |  |  |  |
| Marital Status |  |  | — |  | 0.596 |
| Unmarried | 2(4.3) | 2(8.7) |  |  |  |
| Married | 44(95.7) | 21(91.3) |  |  |  |
| Age at first diagnosis (years) | 32±9.14 | 33±10.02 | 0.424 |  | 0.673 |
| Baseline monthly alcohol consumption (drinks) | 352.6 (237.4, 525.6) | 234.0 (153.9, 465.9) | 1.884 |  | 0.060 |
| Baseline heavy-drinking days (days) | 26.0 (17.0, 28.0) | 24.0 (14.0, 28.0) | 1.012 |  | 0.312 |
| Duration since diagnosis (years) | 9(5, 13) | 10(4, 17) | -0.491 |  | 0.623 |
| Current smokers | 45(97.8) | 21(91.3) | — |  | 0.256 |
| Cigarettes/day among current smokers | 20(10, 20) | 20(10, 20) | -0.340 |  | 0.734 |

Values are presented as mean ± SD, median (IQR), or n (%), as appropriate. Between-group comparisons in the randomized cohort used independent-samples t test (Welch if needed), Mann–Whitney U test, Pearson’s χ² test, or Fisher’s exact test, as appropriate. Abbreviations: NI, naltrexone implant; BMI, body mass index; CNY, Chinese yuan.

**Table S1b. Baseline laboratory parameters in the randomized cohort**

| Measure | Naltrexone implant (NI, n=46) | Placebo (n=23) | Test statistic | *P value* |
| --- | --- | --- | --- | --- |
| AST (U/L) | 20.5(16.0, 26.5) | 20.0(18.0, 30.0) | -0.256 | 0.798 |
| ALT (U/L) | 22.9(16.7, 38.8) | 19.2(15.3, 27.9) | -1.197 | 0.231 |
| GGT (U/L) | 74.8(44.3, 163.6) | 78.3(53.0, 185.7) | -0.452 | 0.651 |
| Total bilirubin (μmol/L) | 7.65(6.10, 11.50) | 8.60(6.70, 10.60) | -0.159 | 0.874 |
| Direct bilirubin (μmol/L) | 2.95±1.22 | 2.89±1.25 | -0.188 | 0.852 |
| Albumin (g/L) | 43.2(40.9, 45.8) | 43.4(40.9, 46.5) | -0.325 | 0.745 |
| Total protein (g/L) | 68.3(65.1, 70.9) | 68.5(64.6, 72.3) | -0.675 | 0.5 |
| Triglycerides (mmol/L) | 1.53(1.18, 2.39) | 1.93(1.59, 3.52) | -1.394 | 0.163 |
| HDL-C(mmol/L) | 1.09(0.89, 1.31) | 1.11(0.95, 1.34) | -0.032 | 0.975 |
| Serum creatinine (μmol/L) | 65.9±9.51 | 66.6±8.86 | 0.313 | 0.755 |
| BUN(mmol/L) | 3.95±1.08 | 4.09±1.21 | 0.474 | 0.637 |
| Uric acid (μmol/L) | 370.5(335.8, 427.5) | 382.0(324.0, 429.0) | -0.115 | 0.909 |
| Potassium (mmol/L) | 4.07±0.32 | 4.00±0.36 | -0.875 | 0.384 |
| Glucose (mmol/L) | 4.31(3.94, 4.57) | 4.32(3.98, 4.58) | -0.14 | 0.889 |

Values are presented as mean ± SD or median (IQR), as appropriate. Between-group comparisons used independent-samples t test (Welch if needed) or Mann–Whitney U test, as appropriate. Abbreviations: AST, aspartate aminotransferase; ALT, alanine aminotransferase; GGT, γ-glutamyltransferase; HDL-C, high-density lipoprotein cholesterol; BUN, blood urea nitrogen.

**Table S1c.Baseline characteristics across the four groups**

| Variable | Naltrexone responders (RN, n=18) | Placebo group（PL, n=10) | Naltrexone non-responders  (NR, n=10) | Healthy controls (HC, n=10) | Test statistic | P value |
| --- | --- | --- | --- | --- | --- | --- |
| Age (years) | 43.7 ± 10.39 | 41.7 ± 9.63 | 40.3 ± 9.7 | 41.7 ± 2.75 | 0.234 | 0.792 |
| Height (cm) | 170.6 ± 7.12 | 172.2 ± 4.92 | 172.0 (164.0, 175.0) | 172.6 ± 4.27 | 0.464 | 0.632 |
| Weight (kg) | 66 (60, 69.8) | 63.8 (55, 73.5) | 63.0 (61.0, 77.0) | 71 (64.3, 74.3) | 2.589 | 0.274 |
| BMI (kg/m²) | 22.6 (21.3, 23.8) | 21.4 (19.9, 23.3) | 22.6 (20.5, 25.1) | 23.4 (22.7, 23.9) | 4.356 | 0.113 |
| Years of education | 13 (11.3, 15) | 15 (12, 16) | 14.5 (12.0, 16.0) | 12 (11, 12.8) | 3.942 | 0.139 |
| Baseline monthly alcohol consumption (drinks) | 369.6 (226.4, 548.1) | 244.4 (177.1, 400.6) | 334.9 (259.2, 705.6) | — | -1.319 | 0.187 |
| Baseline heavy-drinking days (days) | 26.5 (15.8, 28) | 23.5 (13.8, 25.5) | 28.0 (21.0, 28.0) | — | -1.391 | 0.164 |
| Current smokers | 17(94.4) | 10(100) | 10(100) | — | — | 1.000 |
| Cigarettes/day among | 20.0 (10.0, 20.0) | 20.0 (10.0, 20.0) | 20.0 (15.0, 20.0) | — | 0.241 | 0.887 |

Values are presented as mean ± SD, median (IQR), or n (%), as appropriate. P values reflect overall comparisons across the clinical subgroups and were obtained using one-way ANOVA, Kruskal–Wallis test, Pearson’s χ² test, or Fisher’s exact test, as appropriate. Smoking-related variables were available for the clinical subgroups only (RN, NR, and PL). “—” indicates not assessed in healthy controls. For sparse categorical variables, Fisher’s exact test was used and the test statistic is not shown.1 standard drink = 10 g ethanol. Abbreviations: RN, naltrexone responders; NR, naltrexone non-responders; PL, placebo; HC, healthy controls; BMI, body mass index.

**Table S1d. Descriptive baseline characteristics of NI participants included versus not included in the lipidomics subset**

| Variable | NI_lipid (RN+NR) | NI_non_lipid | Test statistic | P value |
| --- | --- | --- | --- | --- |
| Age (years) | 42.5 ± 10.1 | 41.8 ± 9.6 | 0.213 | 0.832 |
| Height (cm) | 170.5 ± 6.4 | 168.7 ± 5.9 | 0.962 | 0.342 |
| Weight (kg) | 65.5 (60.8, 69.8) | 64.0 (55.4, 69.8) | 270.5 | 0.685 |
| BMI (kg/m²) | 22.6 (20.8, 23.8) | 22.5 (20.3, 23.6) | 269 | 0.710 |
| Years of education | 14.0 (12.0, 15.2) | 12.0 (9.0, 14.8) | 309.5 | 0.194 |
| Baseline monthly alcohol consumption (drinks) | 360.2 (255.0, 621.4) | 332.6 (207.0, 454.2) | 294 | 0.350 |
| Baseline heavy-drinking days (days) | 28.0 (19.5, 28.0) | 20.0 (15.5, 27.0) | 340 | 0.041 |
| Current smokers | 27 (96.4) | 18 (100.0) | — | 1 |
| Cigarettes/day among current smokers | 20.0 (10.0, 20.0) | 20.0 (15.0, 20.0) | 198.5 | 0.28 |

Values are presented as mean ± SD, median (IQR), or n (%), as appropriate. P values reflect between-group comparisons between NI_lipid and NI_non_lipid and were obtained using the independent-samples t test, Mann–Whitney U test, Pearson’s χ² test, or Fisher’s exact test, as appropriate. NI_lipid includes naltrexone-implant participants included in the lipidomics subset (RN + NR), whereas NI_non_lipid includes the remaining naltrexone-implant participants not included in the lipidomics subset. Because the lipidomics subset was availability-based and responder-enriched, these comparisons are provided for descriptive interpretation of subset composition.
