# Supplementary material for: A Membrane‐Centric Plasma Lipidomic Signature of Response to Long‐Acting Naltrexone in Alcohol Use Disorder
Source: Addict Biol. 2026 May 12;31(5):e70165. doi: 10.1111/adb.70165 (PMC13167251; doi:10.1111/adb.70165)
Supplement: Supplementary file 5 — Table S2: Primary and secondary outcomes (GEE, exchangeable, robust SE). [file ADB-31-e70165-s008.docx]

**Supplementary Table S2. Primary and secondary outcomes (GEE, exchangeable, robust SE)**

| Time window | NI n | PL n | NI median (IQR), % | PL median (IQR), % | Mann–Whitney U | p value | HL Δ (NI–PL), pp | 95% CI |
| --- | --- | --- | --- | --- | --- | --- | --- | --- |
| Weeks 1–24 (overall, PHDD%) | 46 | 20 | 1.49 (0.00–10.71) | 13.39 (1.79–48.66) | 317 | 0.0415 | -5.95 | -23.21 to 0.00 |

| Model | Effect | df | Wald χ² | P value | Estimate | 95% CI |
| --- | --- | --- | --- | --- | --- | --- |
| Model 1 (HDD, Poisson–log) | Time (wk3, wk6 vs baseline) | 2 | 15.54 | <0.001 | — | — |
| Model 1 (HDD, Poisson–log) | Group × Time | 2 | 7.61 | 0.022 | — | — |
| Model 1 (HDD, Poisson–log) | NI vs PL @ weeks 9–12 (RR) |  |  | 0.456 | 0.72 | 0.30–1.72 |
| Model 1 (HDD, Poisson–log) | NI vs PL @ weeks 21–24 (RR) |  |  | 0.041 | 0.50 | 0.26–0.97 |
| Model 2 (ΔHDD, Gaussian–identity) | Time (wk3, wk6 vs baseline) | 2 | 15.54 | <0.001 | — | — |
| Model 2 (ΔHDD, Gaussian–identity) | Group × Time | 2 | 7.61 | 0.022 | — | — |
| Model 2 (ΔHDD, Gaussian–identity) | NI vs PL @ weeks 9–12 (Δ days) |  |  | 0.299 | -2.74 | -7.91 to 2.43 |
| Model 2 (ΔHDD, Gaussian–identity) | NI vs PL @ weeks 21–24 (Δ days) |  |  | 0.026 | -6.24 | -11.75 to -0.73 |

Panel A: Primary endpoint—PHDD% over weeks 1–24 by group (NI vs PL); values are median (IQR) with Mann–Whitney U, two-sided p, and Hodges–Lehmann difference. Panel B: Longitudinal secondary analysis—GEE with subjects as clusters, exchangeable correlation, and robust SE. Model 1: Poisson–log for HDD counts with offset log (28). Model 2: Gaussian–identity for change from baseline (ΔHDD). Reported are Wald χ²(df), p for time and group×time, and NI vs PL contrasts at weeks 9–12 and 21–24 as RR (Model 1) or Δ days (Model 2) with 95% CI. Abbreviations: NI, naltrexone implant; PL, placebo; PHDD, percentage of heavy-drinking days; HDD, heavy-drinking days; RR, rate ratio; CI, confidence interval.
