# Supplementary material for: A Membrane‐Centric Plasma Lipidomic Signature of Response to Long‐Acting Naltrexone in Alcohol Use Disorder
Source: Addict Biol. 2026 May 12;31(5):e70165. doi: 10.1111/adb.70165 (PMC13167251; doi:10.1111/adb.70165)
Supplement: Supplementary file 7 — Table S4: Exploratory analyses in naltrexone nonresponders (NR). [file ADB-31-e70165-s005.docx]

**Supplementary Table S4. Exploratory analyses in naltrexone non-responders (NR)**

Supplementary Table S4a. Exploratory cross-sectional comparisons between NR and PL at Week 12: Class-level lipid abundances

| Feature | NR mean (log 10) | PL mean (log 10) | Δlog10 (NR-PL) | Fold change (NR/PL) | P value | Q value |
| --- | --- | --- | --- | --- | --- | --- |
| PI | 9.895 | 8.606 | 1.289 | 19.449 | 5.03e-13 | 4.53e-12 |
| PEt | 6.877 | 8.168 | -1.291 | 0.051 | 3.12e-12 | 1.41e-11 |
| LPC | 9.426 | 6.018 | 3.407 | 2554.180 | 1.76e-08 | 5.27e-08 |
| AEA | 6.426 | 7.455 | -1.029 | 0.094 | 5.45e-08 | 1.23e-07 |
| PC | 11.739 | 11.393 | 0.347 | 2.222 | 1.13e-04 | 2.03e-04 |
| Cer | 3.383 | 7.422 | -4.039 | 9.14e-05 | 0.001 | 0.002 |
| PE | 10.308 | 10.139 | 0.169 | 1.476 | 0.148 | 0.190 |
| SM | 9.982 | 9.868 | 0.114 | 1.300 | 0.317 | 0.357 |
| DMPE | 9.279 | 9.259 | 0.020 | 1.047 | 0.806 | 0.806 |

Values are shown on the transformed scale used for statistical testing. Class-level abundances were summed on the linear scale and then log10-transformed. Fold change was calculated as NR/PL on the linear scale. P values were adjusted using the Benjamini-Hochberg false discovery rate procedure.

Supplementary Table S4b. Exploratory cross-sectional comparisons between NR and PL at Week 12: membrane-related lipid indices

| Feature | NR mean | PL mean | Difference (NR-PL) | P value | Q value |
| --- | --- | --- | --- | --- | --- |
| n-3 in PC | -0.605 | -2.789 | 2.184 | 2.24e-10 | 1.34e-09 |
| n-3 in PE | -3.129 | -2.052 | -1.078 | 0.001 | 0.003 |
| AA in PC | -1.629 | -2.334 | 0.705 | 0.037 | 0.075 |
| AA in PE | 2.053 | 1.139 | 0.914 | 0.089 | 0.134 |
| PC/PE | 1.431 | 1.253 | 0.178 | 0.147 | 0.176 |
| DMPE/PE | -1.029 | -0.880 | -0.149 | 0.283 | 0.283 |

Values are shown on the transformed scale used for statistical testing. Ratio indices were analyzed on the log10-transformed scale, whereas proportional indices were analyzed on the logit-transformed scale. P values were adjusted using the Benjamini-Hochberg false discovery rate procedure.

Supplementary Table S4c. Exploratory cross-sectional comparisons between RN and NR at Week 12: membrane-related lipid indices

| Feature | RN mean | NR mean | Difference (RN-NR) | P value | Q value |
| --- | --- | --- | --- | --- | --- |
| n-3 in PC | -0.929 | -0.605 | -0.325 | 0.150 | 0.451 |
| n-3 in PE | -3.051 | -3.129 | 0.078 | 0.777 | 0.932 |
| AA in PC | -1.641 | -1.629 | -0.012 | 0.973 | 0.973 |
| AA in PE | 2.433 | 2.053 | 0.380 | 0.454 | 0.817 |
| PC/PE | 1.746 | 1.431 | 0.315 | 0.015 | 0.093 |
| DMPE/PE | -0.945 | -1.029 | 0.085 | 0.544 | 0.817 |

Values are shown on the transformed scale used for statistical testing. Ratio indices were analyzed on the log10-transformed scale, whereas proportional indices were analyzed on the logit-transformed scale. P values were adjusted using the Benjamini-Hochberg false discovery rate procedure.
