# Supplementary material for: A Membrane‐Centric Plasma Lipidomic Signature of Response to Long‐Acting Naltrexone in Alcohol Use Disorder
Source: Addict Biol. 2026 May 12;31(5):e70165. doi: 10.1111/adb.70165 (PMC13167251; doi:10.1111/adb.70165)
Supplement: Supplementary file 9 — Table S6: Axis‐level statistics for primary contrasts at Week 12. [file ADB-31-e70165-s007.docx]

***Supplementary Table S6. Axis-level statistics for primary contrasts at Week 12***

Supplementary Table S6a. Group-level descriptive statistics for membrane-related lipid indices at Week 12

| Feature | HC mean | PL mean | RN mean |
| --- | --- | --- | --- |
| n-3 in PC | -3.119 | -2.789 | -0.929 |
| n-3 in PE | -2.062 | -2.052 | -3.051 |
| AA in PC | -2.400 | -2.334 | -1.641 |
| AA in PE | 1.434 | 1.139 | 2.433 |
| PC/PE | 1.069 | 1.253 | 1.746 |
| DMPE/PE | -1.073 | -0.880 | -0.945 |

Values are shown on the transformed scale used for statistical testing. Ratio indices were analyzed on the log10-transformed scale, whereas proportional indices were analyzed on the logit-transformed scale. HC, PL, and RN means are reported on the same transformed scales.

Supplementary Table S6b. Primary cross-sectional comparisons between RN and PL at Week 12: membrane-related lipid indices

| Feature | RN mean | PL mean | Difference (RN-PL) | P value | Q value | Hedges' g (95% CI) |
| --- | --- | --- | --- | --- | --- | --- |
| n-3 in PC | -0.929 | -2.789 | 1.860 | 3.66e-09 | 2.19e-08 | 2.78 (1.70 to 3.86) |
| n-3 in PE | -3.051 | -2.052 | -0.999 | 3.72e-06 | 5.58e-06 | -1.88 (-2.81 to -0.95) |
| AA in PC | -1.641 | -2.334 | 0.693 | 0.002 | 0.002 | 1.05 (0.23 to 1.87) |
| AA in PE | 2.433 | 1.139 | 1.294 | 2.15e-07 | 4.29e-07 | 2.47 (1.45 to 3.50) |
| PC/PE | 1.746 | 1.253 | 0.493 | 1.25e-07 | 3.75e-07 | 3.13 (1.98 to 4.29) |
| DMPE/PE | -0.945 | -0.880 | -0.065 | 0.406 | 0.406 | -0.29 (-1.06 to 0.49) |

Values are shown on the transformed scale used for statistical testing. Ratio indices were analyzed on the log10-transformed scale, whereas proportional indices were analyzed on the logit-transformed scale. P values were adjusted using the Benjamini-Hochberg false discovery rate procedure. Hedges' g values with 95% confidence intervals are reported on the same transformed scales used for statistical testing.

Supplementary Table S6c. Primary cross-sectional comparisons between PL and HC at Week 12: membrane-related lipid indices

| Feature | PL mean | HC mean | Difference (PL-HC) | P value | Q value | Hedges' g (95% CI) |
| --- | --- | --- | --- | --- | --- | --- |
| n-3 in PC | -2.789 | -3.119 | 0.330 | 0.021 | 0.043 | 1.14 (0.19 to 2.10) |
| n-3 in PE | -2.052 | -2.062 | 0.010 | 0.954 | 0.954 | 0.02 (-0.85 to 0.90) |
| AA in PC | -2.334 | -2.400 | 0.066 | 0.350 | 0.420 | 0.41 (-0.48 to 1.30) |
| AA in PE | 1.139 | 1.434 | -0.295 | 0.127 | 0.190 | -0.69 (-1.59 to 0.22) |
| PC/PE | 1.253 | 1.069 | 0.184 | 0.007 | 0.024 | 1.32 (0.34 to 2.29) |
| DMPE/PE | -0.880 | -1.073 | 0.193 | 0.008 | 0.024 | 1.29 (0.32 to 2.26) |

Values are shown on the transformed scale used for statistical testing. Ratio indices were analyzed on the log10-transformed scale, whereas proportional indices were analyzed on the logit-transformed scale. P values were adjusted using the Benjamini-Hochberg false discovery rate procedure. Hedges' g values with 95% confidence intervals are reported on the same transformed scales used for statistical testing.

Supplementary Table S6d. Exploratory cross-sectional comparisons between RN and HC at Week 12: membrane-related lipid indices

| Feature | RN mean | HC mean | Difference (RN-HC) | P value | Q value |
| --- | --- | --- | --- | --- | --- |
| n-3 in PC | -0.929 | -3.119 | 2.190 | 3.13e-10 | 9.40e-10 |
| n-3 in PE | -3.051 | -2.062 | -0.989 | 5.26e-05 | 7.89e-05 |
| AA in PC | -1.641 | -2.400 | 0.759 | 8.00e-04 | 9.61e-04 |
| AA in PE | 2.433 | 1.434 | 0.999 | 2.18e-05 | 4.36e-05 |
| PC/PE | 1.746 | 1.069 | 0.677 | 1.57e-12 | 9.44e-12 |
| DMPE/PE | -0.945 | -1.073 | 0.128 | 8.04e-02 | 8.04e-02 |

Values are shown on the transformed scale used for statistical testing. Ratio indices were analyzed on the log10-transformed scale, whereas proportional indices were analyzed on the logit-transformed scale. P values were adjusted using the Benjamini-Hochberg false discovery rate procedure. RN–HC statistics were derived from the reported group-level summary statistics using the same Welch's t-test and BH-FDR framework as described in Methods.
